# BioStructMap: a Python tool for integration of protein structure and sequence-based features

**DOI:** 10.1093/bioinformatics/bty474

**Published:** 2018-06-21

**Authors:** Andrew J Guy, Vashti Irani, Jack S Richards, Paul A Ramsland

**Affiliations:** 1 Life Sciences, Burnet Institute, Melbourne, VIC, Australia; 2 Department of Immunology, Monash University, Melbourne, VIC, Australia; 3 Department of Medicine, University of Melbourne, Melbourne, VIC, Australia; 4 Department of Infectious Diseases, Monash University, Melbourne, VIC, Australia; 5 Victorian Infectious Diseases Service, Royal Melbourne Hospital, Melbourne, VIC, Australia; 6 Department of Surgery, Austin Health, University of Melbourne, Heidelberg, VIC, Australia; 7 School of Science, RMIT University, Bundoora, VIC, Australia

## Abstract

**Summary:**

A sliding window analysis over a protein or genomic sequence is commonly performed, and we present a Python tool, BioStructMap, that extends this concept to three-dimensional (3D) space, allowing the application of a 3D sliding window analysis over a protein structure. BioStructMap is easily extensible, allowing the user to apply custom functions to spatially aggregated data. BioStructMap also allows mapping of underlying genomic sequences to protein structures, allowing the user to perform genetic-based analysis over spatially linked codons—this has applications when selection pressures arise at the level of protein structure.

**Availability and implementation:**

The Python BioStructMap package is available at https://github.com/andrewguy/biostructmap and released under the MIT License. An online server implementing standard functionality is available at https://biostructmap.burnet.edu.au.

**Supplementary information:**

Supplementary data are available at *Bioinformatics* online.

## 1 Introduction

Consideration of three-dimensional (3D) protein structure is important in many areas of research, including antibody-antigen interactions, protein–protein interactions and drug interactions with proteins. For example, antibody recognition of a dominant epitope can lead to selection pressures on residues associated with that epitope; these residues may be distant in the linear protein sequence despite being spatially connected. In immunology, these non-linear sequence-structure relationships are referred to as discontinuous or conformational epitopes. There are a number of pre-existing online tools that allow for visualization and mapping of pre-defined features onto protein structures (Ashkenazy *et al.*, 2010; Baker and Porollo, 2016; Porollo and Meller, 2007; Segura *et al.*, 2017), however none of these tools allow for application of a 3D sliding window over a protein structure using user-defined functions. There are many settings in which sliding window analysis is applied to genomic or protein sequences, and we demonstrate that this sliding window approach can be extended to 3D protein structures.

## 2 Materials and methods

We present here a Python package named BioStructMap that allows mapping of sequence-associated data onto a protein structure. This tool also allows for the application of a 3D ‘sliding window’ over a protein structure. The user can apply a variety of functions to spatially aggregated data, mapping the result back to the central residue within each window. The user must provide sequence-aligned data, a reference sequence and PDB format coordinates over which to process data. For each residue in the structure, all residues within a user-defined radius are selected. Data corresponding to these residues (i.e. specific characteristics of interest for these residues) is then passed to a function that returns a numerical value, which is then mapped back to the central residue (Supplementary Fig. S1). A number of pre-defined functions are included in the BioStructMap package. Users can also supply their own function for data processing. Data is output as a Python dictionary of residues and associated values, written to a PDB file in the B-factor column, or as a text file. Results can be viewed using PyMOL or similar programs.

BioStructMap uses the Biopython Bio.PDB module for handling PDB files, and can accept both PDB and mmCIF files as input. Sequence alignments are performed using either the NCBI BLAST+ package or the Biopython Bio.pairwise2 module. Alignment of DNA sequences to protein sequences is performed using Exonerate (Slater and Birney, 2005) which allows handling of intron-containing sequences and reverse-sense translation. Calculation of Tajima’s D is performed using the Python DendroPy package (Sukumaran and Holder, 2010).

The source code for BioStructMap is available on GitHub (https://github.com/andrewguy/biostructmap) or via the Python Package Index. A simple web-server interface is also available at https://biostructmap.burnet.edu.au, using the JavaScript NGL viewer for visualization of protein structures (Rose and Hildebrand, 2015). Results can be viewed in the browser or downloaded as PDB files. Further details on BioStructMap use are available in Supplementary Material.

## 3 Usage example

**Fig. 1.**
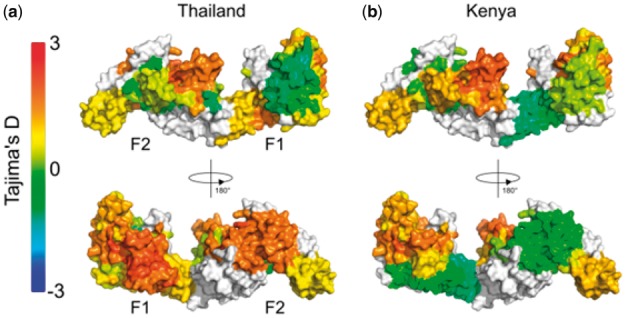
Tajima’s D calculation applied as a 3D sliding window over the protein structure of *P. falciparum* EBA-175 RII. The F1 and F2 domains are indicated on the monomeric structure. Nucleotide sequences were obtained from *P. falciparum* isolates from (**a**) Thailand (*n* = 48) and (**b**) Kenya (*n* = 39) (Verra *et al.*, 2006). The BioStructMap Python package was used to apply Tajima’s D calculations using a 3D sliding window with a radius of 15 Å. The structural model is available via ModBase, accession number: ed998157a605f5e58ed66e198e0ae1ab. Structures were visualized with PyMOL

In areas of endemic malaria, immune selection pressure on the malaria parasite can lead to balancing selection, in which low-frequency alleles are maintained at a higher proportion than would otherwise be expected under a neutral model of selection. Tajima’s D (Tajima, 1989) is one statistic that has been used to identify regions under balancing selection within the malaria genome, and has previously been applied as a sliding window over genes of interest (Arnott *et al.*, 2013, 2014). We have also previously applied the BioStructMap tool to key vaccine candidates from *P. falciparum* and *P. vivax*, incorporating protein structural information into calculations of selection pressures and diversity (Guy *et al.*, 2018a, b). We illustrate here one of the potential uses for the BioStructMap tool, applying a 3D sliding window calculation of Tajima’s D over the protein structure of *Plasmodium falciparum* EBA-175 Region II (RII), a leading malaria vaccine candidate (Fig. 1). This approach groups data that are spatially connected but are distant in the linear sequence. Nucleotide sequences for EBA-175 RII were extracted from GenBank, originally deposited from a study examining signatures of selection in *P. falciparum* strains from Kenya and Thailand (Verra *et al.*, 2006). Since known structures contain a number of unresolved residues, ModPipe (Eswar, 2003) was used to generate a comparative structural model for EBA-175 RII. A radius of 15 Å was selected for each window as this is the typical maximum-dimension for an antibody-antigen interface (Ramaraj *et al.*, 2012). When analyzed, a surface exposed loop with a high spatially derived Tajima’s D value is identified in both Kenyan and Thai isolates. Importantly, this region is involved in the dimerization of EBA-175 RII around its glycophorin A binding partner on the surface of the human red blood cell (Tolia *et al.*, 2005), and antibodies that target the dimerization interface of EBA-175 RII have previously been shown to be highly effective at inhibiting parasite entry into red blood cells (Chen *et al.,* 2013). A region within the F1 domain is also identified as having high Tajima’s D values within Thai samples, but to a much lesser extent in Kenyan samples. Further experimental work would be required to validate this region as a target of functional antibody responses.

## 4 Concluding remarks

The BioStructMap package and associated web interface allow for visualization of sequence-aligned data over a 3D protein structure, as well as allowing the incorporation of protein structural information into sequence-based metrics using a 3D sliding window approach. This tool is applicable to a variety of problems, including identification of regions under various forms of genetic, immunological or drug selection pressure and spatial mapping of residue characteristics that may affect immunogenicity, solubility, binding interaction, etc. The tool is easily extensible, allowing users to define their own functions to apply to spatially aggregated data.

## Supplementary Material

Supplementary Figure S1

Supplementary Data
