# Supplementary material for: BioStructMap: a Python tool for integration of protein structure and sequence-based features
Source: Bioinformatics. 2018 Jun 21;34(22):3942–4. doi: 10.1093/bioinformatics/bty474 (PMC6223362; doi:10.1093/bioinformatics/bty474)
Supplement: Supplementary Figure S1 [file bty474_supplementary_figure_s1.pdf]

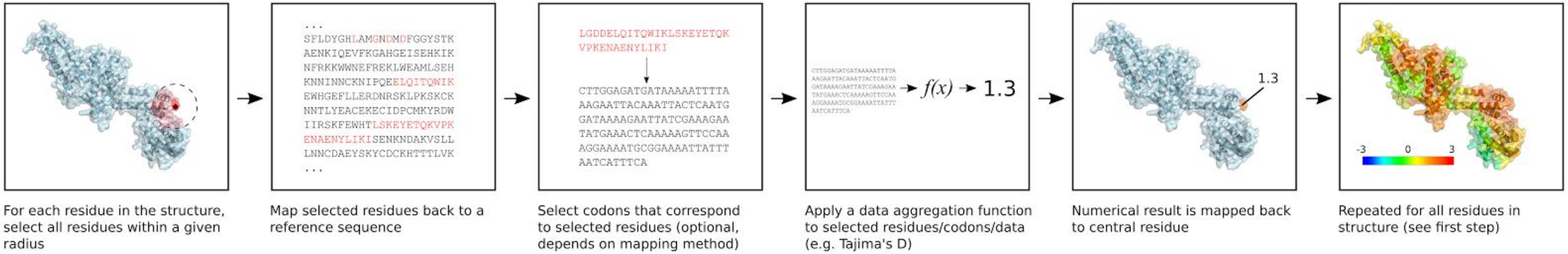

Fig. S1: Application of a 3D sliding window over a protein structure, as implemented in BioStructMap. For each residue in the protein structure, residues within a given radius are identified, and the data corresponding to these residues is extracted. This data is user-supplied, and could be sequence-aligned numerical data, the location of polymorphic residues or genomic sequences. This data is passed to a data aggregation function (e.g. calculate Tajima's D for codons which map to selected residues), and the result from this function mapped back to the central residue. This process is repeated for every residue in the protein structure. BioStructMap is highly flexible with regards to data aggregation functions and user-supplied data—users can write their own data aggregation functions to process specific sequence-aligned data.
