# Supplementary material for: BioStructMap: a Python tool for integration of protein structure and sequence-based features
Source: Bioinformatics. 2018 Jun 21;34(22):3942–4. doi: 10.1093/bioinformatics/bty474 (PMC6223362; doi:10.1093/bioinformatics/bty474)
Supplement: Supplementary Data [file bty474_supplementary_usage_instructions.pdf]

BioStructMap Usage Guide

--- Andrew Guy, 2017

1. Introduction

1.1 Rationale

Often in genomics or protein biology, a statistical test or data aggregation function is applied as a sliding window over a gene or protein sequence. For example, to identify regions under balancing selection, Tajima's D has been applied to the *Plasmodium falciparum* AMA1 gene as a sliding window (Arnott et al. 2014). Similarly, a sliding window analysis can be performed over a protein sequence, often using some amino acid propensity scale, such as the Kyte & Doolittle hydrophobicity scale (Kyte and Doolittle 1982). However, these analyses fail to account for the arrangement of a protein in 3D space. In the case of the Tajima's D analysis of *P. falciparum* AMA1 mentioned before, balancing selection is thought to arise a result of immune selection pressure on this particular antigen. Additionally, it is likely that this immune selection pressure is antibody mediated, and hence the result of interactions between antibodies and the structured antigen. Many of the potential interaction sites in such an interaction involve discontinuous regions of the protein sequence i.e. form a conformational/discontinuous epitope. As a result, using a sliding window over the protein sequence is unlikely to fully capture the selection pressures on complex structural epitopes.

With this in mind, we have proposed the application of a 3D sliding window over a protein structure, analogous to the standard 2D sliding window analysis that is often applied over a protein or gene sequence. This 3D sliding window analysis has been implemented in a Python package called BioStructMap.

1.2 Overview

The BioStructMap tools allows for the application of a 3D sliding window analysis over a protein structure. To achieve this, the user must supply a set of sequence-aligned data and a corresponding reference sequence. This reference sequence is used to map data to the protein structure. A set of 3D windows are then created (one for each residue in the structure) with a user-defined radius, and data from each window is passed to a data aggregation function. The result from this data aggregation function is then mapped back to the central residue within each window. These results can then be viewed over the protein structure using a program such as PyMOL (https://pymol.org). This procedure is analagous to the traditional 2D sliding window analysis that is often performed over a protein or gene sequence, but also captures information on the spatial arrangement of residues in 3D space.

1.3 Availability and installation

The BioStructMap package is available via the Python Package Index (PyPI), which means that installation with `pip` is as simple as:

```
pip install biostructmap
```

Alternatively, the latest source code can be downloaded from GitHub and installed:

```
git clone https://github.com/andrewguy/biostructmap.git biostructmap
cd ./biostructmap
python setup.py install
```

It is recommended that users install `numpy` before installing `biostructmap`.

BioStructMap also has soft dependencies on the NCBI BLAST+ tool (https://www.ncbi.nlm.nih.gov/guide/howto/run-blast-local/) and Exonerate (https://www.ebi.ac.uk/about/vertebrate-genomics/software/exonerate). If you choose not to install these, or don't want to use them, all sequence alignments will be performed using the Biopython `Bio.pairwise2` module. This should work just as well if your reference sequence is reasonably similar to the sequence of the PDB file. If this is not the case, then we suggest that a better approach may be to build a homology model using MODELLER (Webb and Sali 2016) and use this instead of using the poorly aligned PDB structure.

If either BLAST+ or Exonerate are not installed, you should indicate this by setting the relevant flags during BioStructMap usage:

```
import biostructmap

biostructmap.seqtools.LOCAL_BLAST = False
biostructmap.seqtools.LOCAL_EXONERATE = False
```

Some functions within BioStructMap also require installation of the DSSP tool (http://swift.cmbi.ru.nl/gv/dssp/). These include calculation of relative solvent accessibility and secondary structure determination. If you wish to use these functions, you must have DSSP installed.

2. Basic Usage

Although the BioStuctMap package contains several modules, most of these work behind the scenes. The `biostructmap` module should be the only module that needs to be directly used in most cases.

2.1 The Structure class

The main class within the `biostructmap` module is the `Structure` class. This is initialised as such:

```
from biostructmap import biostructmap

my_structure = biostructmap.Structure(pdbfile='./1zrl.pdb', pdbname='1ZRL', mmcif=False)
```

The `pdbfile` argument can be either a string of the file path to the PDB file of interest, while the `pdbname` argument is an optional descriptive string that is used when naming output files. The optional `mmcif` flag is used to indicate if the input file is in mmcif format. If you are using an mmcif file, you would instead run:

```
my_structure = biostructmap.Structure(pdbfile='./1zrl.cif', pdbname='1ZRL', mmcif=True)
```

2.2 Mapping data over a structure

The `Structure` class contains a number of methods, the most important being the `map()` method. This method allows for the mapping of data over a protein structure, with the ability to also apply some sort of spatial aggregation to data. The `map` method takes a number of arguments, the most important of which are `data`, `method`, `ref` and `radius`.

The `Structure.map` method returns a dictionary-like object, which can also be used to write output data to a PDB file or CSV file.

data

The `data` parameter is a dictionary mapping individual chains within the protein structure to relevant data objects. The exact form of the data object will depend on the `method` argument selected. For example, if you are mapping the location of polymorphic residues onto a structure, the data would be a list of polymorpic sites:

```
data = {('A, B'): [1, 34, 56, 77, 120, 121, 125],
        ('C', ): [5, 34, 67, 122]
       }
```

In this example, identical chains `A` and `B` are both assigned the same set of data, whilst the unique chain `C` is assigned another set of polymorphic residues. Note that the given polymorphic sites for each chain are aligned to the reference sequence supplied - see below for more detail.

method

The `method` parameter is either a string representing a method for mapping data (one of a number of pre-defined methods), or a custom function for mapping data (explained in more detail below). For example, to map polymorphic sites onto a protein structure, set `method=snps`.

ref

The `ref` parameter is a set of reference sequences for all chains, and is used to align the user-supplied data to the protein structure. All data supplied via the `data` argument should align to these reference sequences.

For example, with identical chains `A` and `B`, and a unique chain `C`, we would have:

```
ref = {'A': 'KTQEDKL...DJSKJK',
       'B': 'KTQEDKL...DJSKJK',
       'C': 'NAPNLEV...KLWELW'}
       # Note: sequences have been condensed for readability
       # You need to provide the full-length sequence!
```

All data provided in the `data` parameter should align to these sequences.

Also note the subtle difference in the keys needed for the `ref` dictionary vs. the `data` dictionary. The `ref` dictionary should have a sequence provided for every chain being evaluated, with the dictionary key being the *string* identifier for that chain. The `data` dictionary requires a *tuple* of chain identifiers for each related data value. This difference arises so that it is possible to map the same data over multiple chains and subsequently ensure we don't duplicate identical data points that might fall within the same radius (from different chains). This will be discussed in more detail in a later section.

If the supplied data is a genomic multiple sequence alignment, then the provided reference sequence should also be a genomic sequence. In this case `biostructmap` will align this genomic sequence to the protein sequence for the relevant chain in the structure. If a genomic sequence is supplied, then the `map_to_dna` argument should also be set to `True`.

If the `ref` argument is not provided, then the sequences for each chain in the structure are used.

radius

The radius (in Angstrom) over which to select nearby residues for inclusion within each 3D window. This defaults to 15 Angstrom, which is the typical maximum dimension for an antibody epitope. If you simply want to map data to individual residues (eg. to display polymorphic sites on a protein structure), set `radius=0`.

selector

When determining which residues fall within a given radius of a central residue, there are a number of ways in which to compute distances between residues. The default behaviour is to compute the minimum distance between any two atoms in each pair of residues. The `selector` argument allows the user to specify other atoms by which to compute residue distance. By default this argument is `'all'`, which gets all non-heterologous atoms. Other potential options include `'CA'`, `'CB'` etc. If an atom is not found within a residue object, then the selection method reverts to using `'CA'`.

rsa_range

If the user wishes to restrict analysis to residues that fall within a given range of relative solvent accessibility (RSA) values (eg. only surface exposed residues), they can provide a tuple to the `rsa_range` argument. This argument takes a tuple in the form `(minimum, maximum)`, where `minimum` and `maximum` are float values between `0` and `1`.

If any residue falls outside the given range of RSA values, then this residue will ignored in all calculations.

RSA is calculated using the DSSP software. If this is not installed and available on the users PATH, then any attempt to use the `rsa_range` argument will fail, throwing an exception.

map_to_dna

The `map_to_dna` argument is a binary flag to indicate if the reference sequence to be aligned is a DNA sequence. This needs to be set to `True` if the reference sequence is a DNA sequence (e.g. when using the Tajima's D method).

method_params

In order to make `biostructmap` flexible and extensible, the `map` method also takes additional arguments that will be passed to the data aggregation method. These arguments should be provided to the `method_params` argument in a dictionary of keyword arguments (key) and associated values (value).

To provide a concrete example of this, we can consider the `'default_mapping'` method that applies a data aggregation method to sequence-aligned numerical data. By default, this method calculates the mean of all data within each radius. However, we can apply other data aggregation functions (e.g. calculate the median) to each 3D window by passing a `method` argument to the `default_mapping` function:

```
import numpy as np

my_structure.map(..., method_params={'method': np.median})
```

2.3 Basic Usage examples

2.3.1 Mapping polymorphic hotspots

One usage of the biostructmap tool is to determine polymorphic hotspots on a protein structure. This requires the user to provide a list of all polymorphic residues of interest and an associated reference sequence. In this example we have a single-chain structure with the PDB file `1zrl.pdb` and a reference sequence in FASTA format in the file `reference.fasta`. We will use the Biopython `SeqIO` module to read in the reference sequence from file. Polymorphic residues are residues 3, 67, 78, 99, 100, 120 and 121, relative to the reference sequence (where the first residue is number 1).

If we were interested in averaging the number of polymorphisms within a 10 Angstrom radius, we would run:

```
import biostructmap
from Bio import SeqIO

reference_seq = SeqIO.read("reference.fasta", "fasta")

my_structure = my_structure = biostructmap.Structure(pdbfile='./1zrl.pdb',
                                                     pdbname='1ZRL')
hotspots = my_structure.map(data={('A',): [3, 67, 78, 99, 100, 120, 121]}
                            method='snps',
                            ref={'A': reference_seq},
                            radius=10
                           )
```

2.3.2 Amino acid propensity scales

We can also apply a 3D sliding window to calculation of amino acid propensity scales. In this example we will apply the Kyte & Doolittle index of hydrophobicity to the protein structure initialized in the above example. We will also demonstrate how to apply a custom amino acid scale as a 3D sliding window.

We can obtain the Kyte & Doolittle scale from the `Biopython` package:

```
from Bio.SeqUtils import ProtParamData
kd_scale = ProtParamData.kd
```

For the `'aa_scale'` method, the `data` argument should be a dictionary representing the amino acid scale of interest. In this example we will use a window size of 15 Angstrom, and only consider surface exposed residues (RSA > 0.2).

```
mean_hydrophocity = my_structure(data=ProtParamData.kd_scale,
                                 method='aa_scale',
                                 ref={'A': reference_seq},
                                 radius=15,
                                 rsa_range=(0.2, 1)
                                 )
```

To use a custom amino acid propensity scale, we just need to provide a dictionary of numerical values for all amino acids. We will apply the 'relative mutability scale' defined by (Dayhoff, Schwartz & Orcutt, 1978). Again, we are only considering surface exposed residues.

```
relative_mutability = {
  'A': 100, 'R': 65, 'N': 134, 'D': 106, 'C': 20, 'Q': 93,
  'E': 102, 'G': 49, 'H': 66, 'I': 96, 'L': 40, 'K': 56,
  'M': 94, 'F': 41, 'P': 56, 'S': 120, 'T': 97, 'W': 18,
  'Y': 41, 'V': 74
}

mean_mutability = my_structure(data=relative_mutability,
                               method='aa_scale',
                               ref={'A': reference_seq},
                               radius=15,
                               rsa_range=(0.2, 1)
                               )
```

2.3.3 Calculation of Tajima's D

Tajima's D is a statistical test used to determine if a sequence is evolving under non-neutral selection pressure. Here we will apply Tajima's D as a 3D sliding window over our protein structure. We need to supply a multiple sequence alignment, using the `biostructmap.SequenceAlignment` class. The multiple sequence alignment is initially supplied as a FASTA file.

In this case, the reference sequence is taken as the first sequence in the multiple sequence alignment. Note the need to set `map_to_dna=True`.

```
msa = biostructmap.SequenceAlignment('./alignment.fasta', file_format='fasta')
reference_seq = str(msa_data[0].seq)

tajimas_d = my_structure(data={('A',): msa},
                         method='tajimasd',
                         ref= {'A': reference_seq},
                         radius=15,
                         map_to_dna=True
                         )
```

2.3.4 Nucleotide diversity

Nucleotide diversity is a metric that is used to quantify the degree of diversity within a particular window on a gene. We can extend this here to a 3D window over a structure to get a sense of the particular regions of the protein structure that are most diverse within a population (at a genomic level).

Again, we need to supply a multiple sequence alignment.

```
msa = biostructmap.SequenceAlignment('./alignment.fasta', file_format='fasta')
reference_seq = str(msa_data[0].seq)

nucleotide_diversity = my_structure(data={('A',): msa},
                                    method='nucleotide_diversity',
                                    ref= {'A': reference_seq},
                                    radius=15,
                                    map_to_dna=True
                                    )
```

2.3.5 Applying a custom data aggregation function

The `'default_mapping'` method allows the user to apply a custom data aggregation function to data within each window. For example, you could calculate the arithmetic mean of data within a window, calculate the maximum or minimum value within a radius, or apply some other metric to data. We will illustrate with a simple calculation of the maximum data value within a 5 Angstrom window.

Note the use of the additional keyword argument `method_params`, which takes a dictionary of additional parameters to pass to the `default_mapping` method. In this case, `default_mapping` takes the keyword argument `method`, which should be a function that can be used to aggregate a list of data points. This `default_mapping` method can be quite useful when constructing custom mapping procedures!

```
data_values = list(range(1000)) # Just some placeholder data

maximum_values = my_structure(data=data_values,
                               method='default_mapping',
                               ref={'A': reference_seq},
                               radius=5,
                               method_params={
                                 'method': max
                               }
                              )
```

2.4 Results

The results for each mapping call are returned in a dictionary-like object (`DataMap` class - a simple class that extends the `dict` class by adding a couple of additional methods to deal with writing results to files).

The main method that is likely to be used from the `DataMap` object is the `write_data_to_pdb_b_factor` method. This writes all data to the B-factor column of a PDB file, allowing easy visualisation in a program such as PyMOL.

We demonstrate the use of this following a simple calculation of average hydrophobicity (see section 2.3.2).

```
import biostructmap
from Bio import SeqIO
from Bio.SeqUtils import ProtParamData

kd_scale = ProtParamData.kd
reference_seq = SeqIO.read("reference.fasta", "fasta")

mean_hydrophocity = my_structure(data=ProtParamData.kd_scale,
                                 method='aa_scale',
```

```
                                ref={'A': reference_seq},
                                radius=15,
                                rsa_range=(0.2, 1)
                                )

mean_hydrophocity.write_data_to_pdb_b_factor(fileobj='./1ZRL_hydrophocity.pdb')
```

For the `write_data_to_pdb_b_factor` method, the `fileobj` keyword argument can be either an output file name as a string, or a file-like object to write output data to. Additionaly keyword arguments for this method are `default_no_value` and `scale_factor`. The `default_no_value` argument is used to specify the numerical value written to the B-factor column if the value for this residue is `None` (non-numerical values can't be written to the B-factor column). The `scale factor` argument is used to scale output values in situations where they are either too big or small to fit within the B-factor column. For example, it is usually sensible to scale nucleotide diversity values by a factor of 1000 (`scale_factor=1000`).

3. Extending BioStructMap

BioStructMap can be extended by providing custom functions with which to process data within each 3D sliding window. We will briefly discuss the format required for these custom data processing functions.

Each data processing function has the format:

```
def some_method(structure, data, residues, ref, **kwargs):
    ...
    return final_data
```

where `structure` is the parent `biostructmap.Structure` object from which the `map` method has been called, `data` is the `data` argument supplied to the `map` method (no filtering has been applied to this object yet!), `residues` is a list of PDB residues within that particular window, and `ref` is a dictionary mapping PDB residue numbers to reference sequence indices.

3.1 Data aligned to a protein sequence

If the custom method being written needs to deal with data that is aligned to a protein sequence, then the key steps that need to be followed are:

1. From the list of PDB residues within the window, extract the positions of these residues in the corresponding reference sequence.
2. Construct a list of applicable data points, given the list of reference-sequence aligned residues.
3. Perform a data aggregation function over these data points, returning a single value (a single, numerical return value is not absolutely required, although writing data to a PDB file will not be possible if data is non-numerical).

We illustrate these steps with a function to calculate the mean of selected data points:

```
import numpy as np

def calculate_mean(_structure, data, residues, ref, ignore_duplicates=True):
    # Step 1: Get a list of all keys from the data object.
    chains = data.keys()

    # Step 1: Extract position of residues in the reference sequence
    ref_residues = [ref[x] for x in residues if x in ref]

    # Step 1: A little bit more manipulation, as each reference residue is given
    # by a (chain, residue number) tuple, while the data keys are tuples that
    # can contain multiple chains (to enable mapping data between several chains).
    # The list of residues below will look like:
```

```
    # [(('A', 'B'), 1), (('A', 'B'), 2), etc.]
    residues = [(chain, x[1]) for chain in chains
                for x in ref_residues if x[0] in chain]

    # If two separate chains both contain the same data point, and both
    # within the same window, then we might want to de-duplicate this data
    # point, to prevent skewing of the result.
    if ignore_duplicates:
        residues = set(residues)

    # Step 2: Get applicable data points
    data_points = [data[res[0]][res[1]] for res in residues]

    # Step 3: If there is any data that maps to residues within the given
    # window, then we apply some data aggregation method to this data.
    # Note that for this mapping procedure, we define this method to be the
    # arithmetic mean by default.
    if data_points:
        result = np.mean(data_points)
    else:
        result = None
    return result
```

This can then be used by passing this method to the `map` function of a `Structure` object. Note that the data passed to the `map` function should be aligned to the reference sequence.

```
data_values = list(range(1000)) # Just some placeholder data
mean_values = my_structure.map(data={('A',): data_values}
                               method=calculate_mean,
                               ref={'A': reference_seq},
                               radius=15
                              )
```

Alternatively, if the user simply wants to apply an aggregation function to selected data points (as in the above example), then the `default_mapping` method provides a nice interface to perform this via the additional keyword argument `method_params`:

```
data_values = list(range(1000)) # Just some placeholder data
mean_values = my_structure.map(data={('A',): data_values}
                               method='default_mapping',
                               ref={'A': reference_seq},
                               radius=15,
                               method_params={'method': np.mean}
                              )
```

3.2 Genomic multiple sequence alignment data

If the user wants to perform a stastical test or data aggregation on codons that map to residues within each window, then the simplest option is to use the `_genetic_test_wrapper` function defined in the `biostructmap.map_functions` module. This is a simple wrapper function that constructs a multiple sequence alignment from all codons that map to resiudes within a window. For example, to define a test to calculate nucleotide diversity:

Firstly, we define a function to calculate nucleotide diversity from a multiple sequence alignment. We are going to use the DendroPy library to perform this.

```
def diversity_from_dendropy(sequence_alignment):
    # Just make sure the alignment is a string in fasta format.
    if not isinstance(alignment, str):
        data = alignment.format('fasta')
    else:
```

```
        data = alignment

    # If the alignment doesn't exist, then return None.
    if not alignment or len(alignment[0]) == 0:
        return None

    # Construct the relevant DenroPy data structure
    seq = dendropy.DnaCharacterMatrix.get(data=data, schema='fasta')

    # Calculate diversity
    diversity = dendropy.calculate.popgenstat.nucleotide_diversity(seq)

    return diversity
```

Now we can use the `_genetic_test_wrapper` function to pass alignments from each window to the `diversity_from_dendropy` function:

```
from biostructmap.map_functions import _genetic_test_wrapper

def _calculate_nucleotide_diversity(_structure, alignments, residues, ref):
    nucleotide_diversity = _genetic_test_wrapper(_structure, alignments,
                                                 residues, ref,
                                                 diversity_from_dendropy)
    return nucleotide_diversity
```

We can then use this function:

```
msa = biostructmap.SequenceAlignment('./alignment.fasta', file_format='fasta')
reference_seq = str(msa_data[0].seq)

nucleotide_diversity = my_structure(data={('A',): msa},
                                    method=_calculate_nucleotide_diversity,
                                    ref= {'A': reference_seq},
                                    radius=15,
                                    map_to_dna=True
                                    )
```

3.3 Genomic data passed to a command line tool

It is also possible to pass data to a command line tool for processing using the general format outline above.

We firstly need to write a temporary file containing a multiple sequence alignment for each window, and then call our command line tool. In this example we will use 'possum' (https://github.com/jeetsukumaran/possum), which calculates a number of population statistics from a multiple sequence alignment.

```
def call_possum(alignment)
    #Run external tool over sub alignment.
    with tempfile.NamedTemporaryFile(mode='w') as seq_file:
        seq_file.write(alignment)
        # Make sure data is actually written to file.
        seq_file.flush()
        process = subprocess.run(["/opt/bin/possum", "-f", "dnafasta", "-q",
                                  "-v", seq_file.name], stdout=subprocess.PIPE)
    try:
        # Just need to parse the output data to get a numerical value.
        tajd = float(process.stdout.decode().strip().split('\t')[-1])
    except ValueError:
        tajd = None
    return tajd
```

Once we have a function that will process a single muliple sequence alignment, we can wrap this in the

`_genetic_test_wrapper` function:

```
def tajimas_d_from_possum(_structure, alignments, residues, ref):
    result = _genetic_test_wrapper(_structure, alignments, residues, ref,
                                   call_possum)
    return result
```

Fianlly, we can use the final function to map Tajima's D over a structure:

```
msa = biostructmap.SequenceAlignment('./alignment.fasta', file_format='fasta')
reference_seq = str(msa_data[0].seq)

tajimas_d = my_structure(data={('A',): msa},
                         method=tajimas_d_from_possum,
                         ref= {'A': reference_seq},
                         radius=15,
                         map_to_dna=True
                         )
```

4. References

- Arnott,A. et al. (2014) Distinct patterns of diversity, population structure and evolution in the AMA1 genes of sympatric Plasmodium falciparum and Plasmodium vivax populations of Papua New Guinea from an area of similarly high transmission. *Malar. J.*, **13**, 233.
- Kyte,J. and Doolittle,R.F. (1982) A simple method for displaying the hydropathic character of a protein. *J. Mol. Biol.*, **157**, 105–132.
- Dayhoff M.O., Schwartz R.M. and Orcutt B.C. (1978) Atlas of Protein Sequence and Structure *National Biomedical Research Foundation*, **5**, 345-352.
- Webb,B. and Sali,A. (2016) Comparative Protein Structure Modeling Using MODELLER. *Curr. Protoc. Bioinformatics*, **54**, 5.6.1–5.6.37.
